# The Effects of Distributed vs. Condensed Schedule for Robot-Assisted Training with Botulinum Toxin A Injection for Spastic Upper Limbs in Chronic Post-Stroke Subjects

**DOI:** 10.3390/toxins13080539

**Published:** 2021-08-01

**Authors:** Jen-Wen Hung, Yen-Wei Chen, Yi-Ju Chen, Ya-Ping Pong, Wen-Chi Wu, Ku-Chou Chang, Ching-Yi Wu

**Affiliations:** 1 Department of Rehabilitation, Chang Gung Memorial Hospital-Kaohsiung Medical Center, Kaohsiung 83301, Taiwan; hung0702@cgmh.org.tw (J.-W.H.); anita61021@cgmh.org.tw (Y.-J.C.); yaping0707@gmail.com (Y.-P.P.); wendy.wu224@gmail.com (W.-C.W.); 2 School of Medicine, College of Medicine, Chang Gung University, Taoyuan 33302, Taiwan; kcchang@cgmh.org.tw; 3 Department of Occupational Therapy and Graduate Institute of Behavioral Sciences, College of Medicine, Taoyuan 33302, Taiwan; yenwei.chen@nyu.edu; 4 Healthy Aging Research Center, Chang Gung University, Taoyuan 33302, Taiwan; 5 Division of Cerebrovascular Diseases, Department of Neurology, Chang Gung Memorial Hospital-Kaohsiung Medical Center, Kaohsiung 83301, Taiwan; 6 Department of Physical Medicine and Rehabilitation, Chang Gung Memorial Hospital at Linkou, Taoyuan 33305, Taiwan

**Keywords:** robotics, spasticity, stroke, rehabilitation, upper extremity

## Abstract

Robot-assisted training (RT) combined with a Botulinum toxin A (BoNT-A) injection has been suggested as a means to optimize spasticity treatment outcomes. The optimal schedule of applying RT after a BoNT-A injection has not been defined. This single-blind, randomized controlled trial compared the effects of two predefined RT approaches as an adjunct to BoNT-A injections of spastic upper limbs in chronic post-stroke subjects. Thirty-six patients received a BoNT-A injection in the affected upper extremity and were randomly assigned to the condensed or distributed RT group. The condensed group received an intervention of four sessions/week for six consecutive weeks. The distributed group attended two sessions/week for 12 consecutive weeks. Each session included 45 min of RT using the InMotion 2.0 robot, followed by 30 min of functional training. The Fugl-Meyer Assessment, Modified Ashworth Scale, Wolf Motor Function Test, Motor Activity Log, and Stroke Self-Efficacy Questionnaire were assessed at pre-training, mid-term, post-training, and at 6 week follow-up, with the exception of the Motor Activity Log, which did not include mid-term measures. After the intervention, both groups had significant improvements in all outcome measures (within-group effects, *p* < 0.05), with the exception of the Wolf Motor Function Test time score. There were no significant differences between groups and interaction effects in all outcome measures. Our findings suggest that RT provided in a fixed dosage as an adjunct to a BoNT-A injection has a positive effect on participants’ impairment and activity levels, regardless of treatment frequency. (ClinicalTrials.gov: NCT03321097).

## 1. Introduction

Improving upper limb function impaired due to a stroke is critical in rehabilitation because the deficit can impact patients’ activities of daily living (ADL). Spasticity is one of the major contributing factors to upper limb dysfunction. Studies have shown that patients with spasticity have greater levels of disability, poorer quality of life, and more caregiver burden than patients without spasticity [1,2,3]. Thus, the treatment of spasticity, particularly of the upper extremity (UE), is an important issue in post-stroke rehabilitation.

Botulinum toxin A (BoNT-A) injection is most widely used for managing focal spasticity. A systematic review and meta-analysis showed robust evidence that BoNT-A has favorable effects on resistance to passive movement and on self-care, but evidence is lacking on the effects on arm-hand capacity [4]. Restoration of UE function is a major goal in stroke rehabilitation even during the chronic phase. Therefore, adding a rehabilitation program after BoNT-A injection was suggested as a means to optimize spasticity treatment outcomes [5]. Bakheit et al. indicated that overall rehabilitation is likely to be more important in producing functional change than a single specific intervention such as BoNT-A injection [6]. Although the benefits of rehabilitative trainings after BoNT-A injection are generally accepted, agreement on the most effective approach is lacking [7].

In addition to conventional rehabilitation training, robot-assisted training (RT), which provides task-specific, high-repetition movement training, has been proved to be effective in patients with moderate and severe arm weakness after stroke [8]. BoNT-A and RT, which appears to be a reasonable synergistic combination, has been evaluated in several studies of patients with stroke [9,10,11,12]. The current evidence is not sufficient to support RT as an adjunctive therapy with BoNT-A, but this is an important area for further research because of the increasing use of RT in stroke rehabilitation. We planned to address a practical issue of the optimal schedule of applying RT after a BoNT-A injection.

Spasticity management with BoNT-A creates a transient plastic state of the neuromotor system that allows motor relearning and recovery [13]. Therefore, pharmacological activity of BoNT-A on spastic muscle is considered crucial for providing a rehabilitation intervention after the injection. The pharmacological effect of BoNT-A commences at 2-to-4 days after the injection, with an expected peak effect at 3 weeks, and its efficacy persists for 6 weeks and up to 9–12 weeks [14,15,16]. When RT is provided in a fixed dosage, it is unknown whether the RT program should be condensed within the most effective period (6 weeks after injection), or distributed across the whole possible effective period of BoNT-A (12 weeks after injection). Previous studies [9,10,11] provided RT for 2 to 5 weeks after the BoNT-A injection, with heterogeneous training frequency and a total dose. No study to date has extended the RT course until the time when the BoNT-A effect may disappear.

To define the optimal schedule for RT as an adjunct therapy with BoNT-A, the effects of different training frequencies on muscle tone and motor function should be considered.

The effect of RT on muscle tone remains uncertain. Gandolfi et al. [17] presented a positive effect on the Modified Ashworth scale (MAS), Bertani and Melegari [18] found no change, whereas Veerbeek and Langbroek-Amersfoort [19] showed RT had a negative effect on muscle tone. Scant information is available for the additive effect of RT when combined with BoNT-A. Saita et al. [11] found the combination therapy significantly improved spasticity after 2 weeks of RT intervention (10 times per week), but the improvement was not statistically significant at the 4 month follow-up. Gandolfi et al. [9] found 5 weeks of RT (two times per week) was as effective as conventional rehabilitation training on muscle tone reduction when combined with BoNT-A. They suggested further research should define the ideal training protocols of RT as an adjunct therapy with BoNT-A.

Regarding motor function, the training schedule may influence skills acquisition and retention [20]. The interval between training sessions has a substantial effect on the learning of motor skills. A distributed schedule, where periods of practice are interspersed with periods of rest, has been demonstrated to benefit motor learning compared with a mass schedule [21]. This superiority may result from the spacing effect [22,23]. Motor functional gains from rehabilitation training involve procedural memory acquisition [24]. Relatively long intervals (days) between practice sessions may facilitate recollection of the motor skill [25], because the greater distribution of training allows for more intervening nights of sleep, during which procedural memory is consolidated [26].

Therefore, this study planned to compare the short- and long-term effects between condensed and distributed RT programs as an adjunct to BoNT-A injections of the spastic upper limb in chronic post-stroke subjects. We hypothesized that subjects who received a distributed RT program would have more improvement in upper limb functional performance and a similar spasticity reduction compared with those who received a condensed RT program.

## 2. Results

We screened 40 subjects for eligibility; of these, 36 met the inclusion criteria, with 18 each randomized to the condensed or the distributed groups. All participants completed the study protocol according to the randomization group. Descriptive characteristics of participants are presented in Table 1. The demographic and clinical characteristics of participants in the two groups did not differ significantly. The UE muscles that were injected and the dose of BoNT-A administered was similar between the two groups (condensed: 336.94 IU; distributed: 338.33 IU; *p* = 0.85).

There were significant within-group effects in all outcome measures, except the WFMT-Time score (*p* = 0.1). There were no significant between-group effects and no interaction effects in all outcome measures. The pairwise comparisons between any two time points for each outcome measure were as follows:

The FMA and SSEQ both showed a significant difference between pre-treatment (*p* = 0.002) and mid-term (*p* = 0.026), between pre-treatment and post-treatment (both *p* < 0.001), and between pre-treatment (*p* = 0.001) and follow-up (*p* = 0.007). No differences were found between any two time points at mid-term, post-treatment, or follow-up.

The MAS-proximal showed a significant difference between pre-treatment and mid-term (*p* < 0.001), between pre-treatment and post-treatment (*p* = 0.008), between mid-term and post-treatment (*p* = 0.002), and between mid-term and follow-up (*p* < 0.001). The MAS-distal showed a significant difference between any two time points (*p* < 0.05), with the exception of a trend showing a difference between mid-term and post-treatment (*p* = 0.058).

The WFMT-Function score only showed a significant difference between pre-treatment and post-treatment (*p* = 0.018), and a difference in the trend between pre-treatment and follow-up (*p* = 0.065).

**Figure 1:**
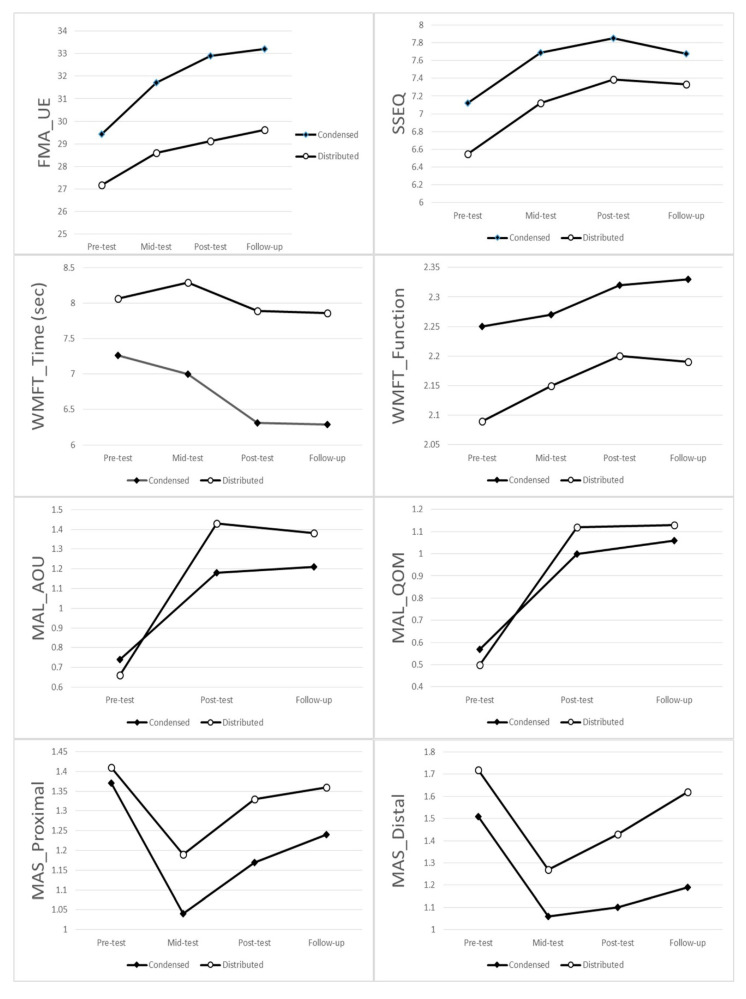
Group means for each assessment time for each outcome measures. Abbreviation: FMA_UE = upper limb subtest of the Fugl-Meyer assessment, SSEQ =Stroke Self-Efficacy Questionnaires, WMFT Time = Wolf Motor Function Test time score, WMFT Function = Wolf Motor Function Test function score, MAL AOU = Motor Activity Log Amount of use score, MAL QOM = Motor Activity Log Quality of movement score, MAS Proximal = Modified Ashworth scale proximal flexors, MAS Distal = Modified Ashworth scale distal flexors.

MAL-AOU and MAL-QOM both showed a significant difference between pre-treatment and post-treatment (both *p* < 0.001), and between pre-treatment and follow-up (both *p* < 0.001) (Figure 1; Table 2 and Table 3).

## 3. Discussion

This is the first randomized controlled study to compare the effects of the two predefined frequencies of the RT approaches as an adjunct to BoNT-A injection of spastic UEs in chronic post-stroke subjects. We found that with a fixed number of training sessions, the RT programs in two different frequencies, either condensing to the peak effective period of BoNT-A or distributing across the whole effective period, resulted in similar gains in the body function and activity/participation domains. Most benefits could be maintained until 6 weeks after training.

RT has exhibited positive effects of reducing motor impairments in patients with moderate and severe arm weakness after a stroke [8]. Moreover, RT may improve convenience and lower the labor cost; a VA Robotics study demonstrated that the total costs (including therapy and healthcare costs) were not greater for the RT than for the usual care [27]. Hence, we suspect RT combined with BoNT-A might optimize the outcome of post-stroke spasticity management. Several studies reported the benefits of this combination therapy [9,10,11,12]; however, the evidence is still not sufficient. Our results provide clinical information for scheduling RT as an adjunctive therapy with BoNT-A injection for post-stroke spasticity.

Our results did not support our original hypothesis that subjects who received a distributed RT would have more improvement in upper limb functional performance compared with those who received a condensed RT. There are some explanations. Previous studies of intervention frequency usually compared the effect of the training sessions within a day vs. across days [21,28]. No rehabilitation therapy conforms to traditional definitions of massed practice because the rehabilitation training sessions are usually conducted over multiple days. Our study therefore focused on whether the effect of the RT program is optimized by the condensed or distributed treatment frequency. The contrast between the two frequencies (four sessions vs. two sessions per week) may be too small to make a difference. In addition, there is evidence that the benefits of distributed learning are relatively less in older adults than in younger adults [29]. Most participants in our study were older adults. Furthermore, the interval from post-training to follow-up was only 6 weeks, which may be too short to allow for differential attenuation of gains in the condensed treatment group.

Highly intensive treatment protocols are an emerging service delivery model in rehabilitation [30,31]. When considering the temporary antispastic effect of BoNT-A, clinicians have usually suggested condensing the post-injection rehabilitation training so that it occurs during the peak effective period of BoNT-A [32]. Our results, however, showed the condensed and distributed treatment protocols of RT both resulted in beneficial effects on measures across impairment and activity/participation domains. Condensed treatment protocols may not be clinically suitable for many patients because of transportation factors or health care services’ availability, or both. A distributed therapy model, such as that used in our study, presents an efficacious and potentially more feasible model of rehabilitation training.

The efficacy of BoNT-A is temporary, up to 9–12 weeks after injection [14,15,16]. It would be interesting to know whether the adjunct therapy to BoNT-A can boost the effect of BoNT-A [33]. An animal study [34] showed that after the injection of BoNT-A, the neurotransmission can be gradually restored by functional rehabilitation of intoxicated motor nerve terminals. Because RT involves massive repetitive movements, some participants worried the massive active movements involving flexor muscles might shorten the antispastic duration of BoNT-A. In our study, the time course of the spasticity reduction in both groups was similar: the decrease in spasticity was greatest at mid-intervention and then declined gradually. This finding supported our hypothesis that treatment frequency of RT would not have different impact on the antispastic effect of BoNT-A. Although the antispastic duration at the distal UE seemed longer than at the proximal UE (the antispastic effect was maintained at the distal UE until the follow-up period but not at the proximal UE), we could not make further inferences because of the limitation of our study design. The aim of this study was to compare the treatment effects between two schedules of RT programs; thus, we did not include a control group without RT to examine whether RT modulates the antispastic effect of BoNT-A. Future study is needed.

Although we did not find differences between the two treatment interval programs in UE functional performance, we confirmed that combining BoNT-A and RT can enhance UE capacity and use. Both groups in our study had significant improvement in FMA at mid-intervention and then maintained the gains until the follow-up period. This finding was in line with other MIT-Manus robotic studies for patients with moderate to severe upper limb functional limitation resulting from stroke [35,36]. However, the effects of RT in UE function or ADL were inconsistent. The VA Robotics Trial found that RT reduced upper limb impairment (FMA motor subscale) and that this advantage translated into significant upper limb functional improvements (WMFT) [35]. Nevertheless, the RAULS study found the improvement in the FMA-UE motor subscale after RT did not translate into improvement in upper limb function or in ADL [36]. Both groups in our study had significant improvement in the quality of UE functional movement, as shown in the WFMT function score, and in ADL, as shown in the MAL-AOU and MAL-QOM after the intervention. This probably occurred because we provided functional task training after RT at each training session.

Because the pharmacological activity of BoNT-A is temporary, sequential outcome evaluations are important to determine the time at which the functional improvement began, was most recognized and, then, probably declined. We performed four assessments (at pre-training, mid-term, post-training, and 6 week follow-up), which allowed us to monitor the sequences of improvements in different functional domains. We found the improvement in body function (FMA) occurred earlier than the improvement in activities (WFMT). Patients also had significantly higher personal confidence to perform ADL soon at the mid-term period. The time lag with motor function improvement after a BoNT-A injection is well known, because patients need to readjust to the decreased muscle tone [37]. The RT probably had a direct effect in enhancing UE movement; therefore, patients may have had improvement in UE movements soon at the mid-term period. When patients perceive that movement has improved, they can also have more confidence to try to perform ADL. Hence, there was significant improvement in SSEQ soon at the mid-intervention period. However, a longer time is required to be proficient in accomplishing more skillful activities.

### Limitations

Some limitations of the study should be considered. First, because it was a controlled study with RT sessions, the study durations of the two groups were different, and we do not know whether both groups had similar outcomes at the end of the entire study (19 weeks after the BoNT-A injection).

Second, although we used several clinical scales to measure functional improvement in accordance with the International Classification of Functioning, Disability and Health framework, these clinical measures may have less sensitivity to change, and scant information for the underlying training effects on motor control was available compared with the instrumental assessments.

Third, we did not recruit a group that did not have RT to show that the effect of change was not only due to the BoNT-A and standard of care alone.

Fourth, we did not control the intensity of other routine stroke rehabilitations (e.g., physical therapy or speech therapy) that did not involve UE training. Although the physical therapy did not involve UE training, this could be a confounder in that patients may theoretically have more physical therapy than another group outside the research, and this may have affected results.

Fifth, we did not assess the cost effectiveness of using RT as an adjunct therapy with BoNT-A treatment. The cost effectiveness of such combination intervention is important and deserves further plentiful research. 

Future research should recruit a larger sample size to include an additional group only with BoNT-A and usual care, use surface electromyography or kinematic analysis for more detailed assessments, conduct assessments for all participants at the end of the entire study, and compare the cost effectiveness of each intervention.

## 4. Conclusions

Our results provide clinicians with information about scheduling RT as an adjunct therapy with BoNT-A injections of a spastic upper limb in chronic moderate-severe post-stroke subjects. RT provided in a fixed dosage combined with BoNT-A had a positive effect on participants’ impairment and activity levels regardless of treatment frequency. If limited service resources are available, the distributed RT program after a BoNT-A injection may be a feasible and effective scheme.

## 5. Materials and Methods

### 5.1. Participants

Participants with stroke were recruited from the rehabilitation department of a tertiary referral hospital. The Institutional Review Board for Human Studies approved the protocol (approval code 201601931A3 on 25 January 2017), and all participants gave informed consent. The inclusion criteria were (1) clinical and imagining diagnosis of a first or recurrent unilateral stroke of ≥6 months; (2) UE spasticity (at least 1 UE muscle with modified Ashworth scale of ≥1+; (3) initial motor part of the Fugl-Meyer Assessment for Upper Extremity (FMA-UE) score ranging from 13 to 56, indicating moderate to severe movement impairment; (4) Mini Mental State Exam score >20, indicating no serious cognitive impairment; and (5) age ≥18 years. The exclusion criteria were (1) bilateral hemispheric or cerebellar lesions; (2) severe aphasia; (3) significant visual field deficits; (4) treatment with BoNT-A ≤4 months before recruitment; or (5) history of orthopedic or other neurologic diseases or medical conditions that would prevent adherence to the rehabilitation protocol.

### 5.2. Randomization

A computerized (block) randomization scheme was used to randomize participants. To minimize possible confounding effects of upper limb motor ability and stroke duration, we stratified participants into groups based on stroke duration (<1 year or ≥1 year) and upper limb motor function (FMA UE score of 17–38 or a FMA UE score of 39–56). Randomization was undertaken in 4 blocks, and each block randomization scheme was within each stratum. A web-based randomization tool [38] (freely available at https://www.randomizer.org/) was used to derive the random table of each stratum by an assistant, who was not involved in the other study procedures. Based on the random table of the stratum, the assistant decided the new participants’ group allocation when they finished the baseline assessment and informed the therapist to conduct their intervention.

### 5.3. Interventions

After a baseline assessment, participants received a BoNT-A injection for their UE spasticity by 1 or 2 senior rehabilitation physicians. Doses and muscles selected for the BoNT-A injection were individualized according to the spasticity patterns and severity of spasticity. Botox brand BoNT-A Purified Neurotoxin Complex (Allergan, an AbbVie Company, Irvine, CA, USA) was used in this study. Location of the targeted muscle was confirmed by echo guidance. Concurrent use of drugs having muscle relaxant properties was maintained at a constant dosage throughout the study. All other routine stroke rehabilitations (e.g., physical therapy or speech therapy) that did not involve UE training proceeded as usual.

One week after the BoNT-A injection, all participants began the 24 training sessions. Participants were assigned to a condensed (4 sessions/week for 6 consecutive weeks) or distributed (2 sessions/week for 12 consecutive weeks) intervention group as determined by stratified randomization based on upper limb motor function (FMA-UE score of 13–40 or FMA-UE score of 41–56). Each training session included 45 min of RT using the InMotion 2.0 robot (Interactive Motion Technologies Inc., Watertown, MA, USA), followed by 30 min of functional training.

### 5.4. RT Procedures

The InMotion 2.0 robot has 3 movement patterns with 3 degrees of freedom: (1) shoulder flexion/extension and abduction/adduction and elbow flexion/extension; (2) forearm pronation/supination; and (3) wrist circumduction, including wrist flexion/extension and abduction/adduction. Our intervention protocol included 160 to 208 repetitions of passive-stretch tasks, 16 to 48 repetitions of the affected arm actively performing tasks, and 80 to 160 repetitions of adaptive robot-assisted tasks.

The functional training included 2 types of tasks: those that simulate robot-training movements, for example, taking a book from a shelf and opening the book (forearm pronation/supination), or using a key and turning a door knob (wrist circumduction); and those are not like robot training movements, such as using spoon for eating.

### 5.5. Outcome Measures

We used clinical assessments to evaluate therapeutic effects of RT in accordance with the International Classification of Functioning, Disability and Health framework published by the World Health Organization World Health Assembly. Clinical assessments for body function and structures included the FMA-UE and Modified Ashworth Scale (MAS). Clinical assessments for activity and participation measures included the Wolf Motor Function Test (WMFT) and Motor Activity Log (MAL). In addition, we used the Stroke Self-Efficacy Questionnaire (SSEQ) to measure individual confidence for functional performance and aspects of self-management.

Evaluators were blind to group allocation. Clinical assessments were performed 4 times: before the intervention (pre-treatment), after the completion of 12 sessions of RT (mid-term), after the completion of 24 sessions of RT (post-treatment), and 6 weeks after the end of RT (follow-up), with the exception of the MAL, which did not include mid-term measures.

### 5.6. Body Function and Structures Measures

Fugl-Meyer Assessment for Upper Extremity (FMA-UE): The FMA-UE was used to assess the patient’s reflexes, movements, and coordination of upper limbs. It consists of 33 items scored on a 3 point ordinal scale (0, cannot perform; 1, performs partially; 2, performs fully) [39]. The total score ranges from 0 to 66, and a higher score indicates better motor function. Satisfactory psychometric properties of the FMA have been demonstrated [40].

Modified Ashworth Scale (MAS): Spasticity of skeletal muscle in UE was evaluated using the MAS assessment, which has shown good reliability and validity [41,42]. For statistical analysis, 1+ was recorded as a score of 1.5. In addition, we estimated the mean of MAS scores in finger flexors, the thumb flexor, and the wrist flexor of each participant as the MAS of the distal UE, and the mean of MAS scores in the shoulder adductor, shoulder internal rotator, elbow flexor, and forearm pronator as the MAS of the proximal UE for further analysis.

### 5.7. Activity and Participation Measures

Wolf Motor Function Test (WMFT): The WMFT is a quantitative measure of UE motor ability through timed and functional tasks [43]. The WMFT includes 17 tasks. Performances were timed and rated using a 6 point ordinal scale. The WMFT has good interrater reliability and criterion validity in patients with UE hemiparesis [44].

Motor Activity Log (MAL): The MAL is a semi-structured interview for stroke patients to assess the amount of use (MAL-AOU) and quality of movement (MAL-QOM) of their affected UE during 30 ADL [45]. The score of each item ranges from 0 to 5, and higher scores represent more frequently used or higher quality of movement. The MAL has established reliability, validity, and responsiveness in patients with stroke [45,46,47].

Stroke Self-Efficacy Questionnaire (SSEQ): The SSEQ is a 13 item self-reported measure that rates participants’ confidence to perform ADL using a 0 (not at all confident) to 10 (very confident) scale. The SSEQ has good validity and feasibility for use in the recovery period after stroke [48].

### 5.8. Statistical Analysis

We used χ^2^ and independent-sample *t* tests to compare participants’ baseline characteristics between groups. To examine the therapeutic effects of the RT, we used mixed analysis of variance to test the differences within groups across 4 measurement times (time effect), to test the differences between groups (condensed vs. distributed), and to test the interaction effect between time and group. Pairwise comparison with Bonferroni adjustments was used to examine the differences between measurement time points. The statistical tests were performed using SPSS 25 software (IBM Corp, Armonk, NY, USA) at the α = 0.05 level of significance.

## Figures and Tables

**Table 1:** Descriptive characteristics of condensed and distributed intervention group.

Variables	Condensed Group (*n* = 18)	Distributed Group (*n* = 18)	*p*
Sex (Male/Female)	14/4	13/5	0.7
lesion side (left/right)	8/10	11/7	0.32
Age (years)	51.73 (13.21)	53 (8.27)	0.73
Type (infarction/hemorrhage)	13/5	9/9	0.17
Time since stroke (months)	38.89 (21.12)	29.11 (25.83)	0.22
Number of strokes (1st time/ 2nd time)	16/2	17/1	0.55
Height (cm)	167.72 (6.65)	165.36 (8.59)	0.36
Weight (kg)	71.72 (2.9)	72.94 (2.99)	0.77

Data are mean (SD). An independent-sample *t* test was used for continuous data, and χ^2^ test was used for categorical data.

**Table 2:** Descriptive statistics for clinical outcome measures.

Outcome Measure	Pre-Test		Mid-Test		Post-Test		Follow-Up	
	Condensed	Distributed	Condensed	Distributed	Condensed	Distributed	Condensed	Distributed
FMA_UE	29.44 (7.95)	27.17 (7.52)	31.72 (8.28)	28.61 (8.07)	32.89 (8.35)	29.11 (7.44)	33.22 (8.48)	29.61 (8.28)
SSEQ	7.12 (1.35)	6.55 (1.44)	7.69 (1.31)	7.12 (1.05)	7.85 (1.43)	7.39 (1.06)	7.68 (1.77)	7.33 (1.17)
WMFT Time	7.26 (2.61)	8.06 (3.62)	7.00 (2.68)	8.29 (3.10)	6.31 (2.29)	7.89 (3.24)	6.29 (2.36)	7.86 (3.10)
WMFT Function	2.25 (0.48)	2.09 (0.40)	2.27 (0.51)	2.15 (0.43)	2.32 (0.48)	2.20 (0.39)	2.33 (0.43)	2.19 (0.37)
MAL AOU	0.74 (0.44)	0.66 (0.48)	N/A		1.18 (0.59)	1.43 (0.78)	1.21 (0.67)	1.38 (0.85)
MAL QOM	0.57 (0.37)	0.50 (0.39)	N/A		1.00 (0.47)	1.12 (0.66)	1.06 (0.54)	1.13 (0.73)
MAS Proximal	1.37 (0.41)	1.41 (0.45)	1.04 (0.49)	1.19 (0.51)	1.17 (0.43)	1.33 (0.51)	1.24 (0.49)	1.36 (0.55)
MAS Distal	1.51 (0.44)	1.72 (0.49)	1.06 (0.55)	1.27 (0.56)	1.10 (0.55)	1.43 (0.50)	1.19 (0.60)	1.62 (0.38)

Data are mean (SD). Abbreviation: FMA_UE = upper limb subtest of the Fugl-Meyer assessment, SSEQ = Stroke Self-Efficacy Questionnaire, WMFT Time = Wolf Motor Function Test time score, WMFT Function = Wolf Motor Function Test function score, MAL AOU = Motor Activity Log Amount of use score, MAL QOM = Motor Activity Log Quality of movement score, MAS Proximal = Modified Ashworth scale proximal flexors, MAS Distal = Modified Ashworth scale distal flexors.

**Table 3:** Inferential statistics for clinical outcome measures.

Outcome Measure	Effect	F (*df*)	*p*
FMA_UE	Within-group	15.59 (3,102)	<0.001
	Between-groups	1.49 (1,34)	0.23
	Time × group	0.93 (3,102)	0.43
SSEQ	Within group	11.04 (3,102)	<0.001
	Between groups	1.45 (1,34)	0.23
	Time × group	0.25 (3,102)	0.86
WMFT Time	Within group	2.12 (3,102)	0.10
	Between groups	2.17 (1,34)	0.15
	Time × group	0.65 (3,102)	0.59
WMFT Function	Within group	4.88 (3,102)	0.003
	Between groups	0.88 (1,34)	0.35
	Time × group	0.24 (3,102)	0.87
MAL AOU	Within group	46.74 (2,68)	<0.001
	Between groups	0.35 (1,34)	0.56
	Time × group	2.77 (2,68)	0.07
MAL QOM	Within group	46.20 (2,68)	<0.001
	Between groups	0.05 (1,34)	0.82
	Time × group	0.76 (2,68)	0.47
MAS Proximal	Within group	16.069 (3,32)	<0.001
	Between groups	0.578 (1,34)	0.452
	Time × group	0.802 (3,32)	0.455
MAS Distal	Within group	26.58 (3,102)	<0.001
	Between groups	3.45 (1,34)	0.07
	Time × group	1.85 (3,102)	0.14

Abbreviation: FMA_UE = upper limb subtest of the Fugl-Meyer assessment, SSEQ =Stroke Self-Efficacy Questionnaires, WMFT Time = Wolf Motor Function Test time score, WMFT Function = Wolf Motor Function Test function score, MAL AOU = Motor Activity Log Amount of use score, MAL QOM = Motor Activity Log Quality of movement score, MAS Proximal = Modified Ashworth scale proximal flexors, MAS Distal = Modified Ashworth scale distal flexors.

## Data Availability

The data presented in this study are available on request from the corresponding author. The data are not publicly available due to ethical issue.

## Suppliers

InMotion 2.0 robot: Bionik Laboratories Corp. 483 Bay Street Office N105 Toronto, ON M5G 2C9 Canada.

## Abbreviations

ADL	activities of daily living
BoNT-A	Botulinum toxin A
FMA-UE	Fugl-Meyer Assessment for Upper Extremity
MAL	Motor Activity Log
MAL-AOU	Motor Activity Log -amount of use
MAL-QOM	Motor Activity Log -quality of movement
MAS	Modified Ashworth Scale
MMSE	Mini Mental State Exam
RT	robot-assisted training
SSEQ	Stroke Self-Efficacy Questionnaire
UE	upper extremity
WMFT	Wolf Motor Function Test
